# Early Colonization of Weathered Polyethylene by Distinct Bacteria in Marine Coastal Seawater

**DOI:** 10.1007/s00248-019-01424-5

**Published:** 2019-08-29

**Authors:** Gabriel Erni-Cassola, Robyn J. Wright, Matthew I. Gibson, Joseph A. Christie-Oleza

**Affiliations:** 1 School of Life Sciences, University of Warwick, Coventry, CV4 7AL UK; 2 Department of Chemistry, University of Warwick, Coventry, CV4 7AL UK; 3 Warwick Medical School, University of Warwick, Coventry, CV4 7AL UK

**Keywords:** Marine plastic pollution, Microbial colonization, Plastisphere, Early biofilm, Weathered polyethylene

## Abstract

Plastic debris in aquatic environments is rapidly colonized by a diverse community of microorganisms, often referred to as the “Plastisphere.” Given that common plastics are derived from fossil fuels, one would expect that Plastispheres should be enriched with obligate hydrocarbon-degrading bacteria (OHCB). So far, though, different polymer types do not seem to exert a strong effect on determining the composition of the Plastisphere, and putative biodegrading bacteria are only found as rare taxa within these biofilms. Here, we show through 16S rRNA gene sequencing that the enrichment of a prominent OHCB member on weathered and non-weathered polyethylene only occurred at early stages of colonization (i.e., after 2 days of incubation in coastal marine water; 5.8% and 3.7% of relative abundance, respectively, vs. 0.6% on glass controls). As biofilms matured, these bacteria decreased in relative abundance on all materials (< 0.3% after 9 days). Apart from OHCB, weathered polyethylene strongly enriched for other distinct organisms during early stages of colonization, such as a specific member of the *Roseobacter* group and a member of the genus *Aestuariibacter* (median 26.9% and 1.8% of the community, respectively), possibly as a consequence of the availability of short-oxidized chains generated from weathering. Our results demonstrate that Plastispheres can vary in accordance with the weathering state of the material and that very early colonizing communities are enriched with taxa that can potentially degrade hydrocarbons. Given the lack of persistent enrichment and overall community convergence between materials over time, common non-hydrolysable polymers might not serve as an important source of carbon for mature Plastispheres once the labile substrates generated from weathering have been depleted.

**Electronic supplementary material:**

The online version of this article (10.1007/s00248-019-01424-5) contains supplementary material, which is available to authorized users.

## Introduction

Recent years have seen heightened societal concern about the abundance and impacts of plastic debris in the marine environment [1, 2]. Being highly recalcitrant materials, plastics accumulate in the environment polluting sediments and surface seawater around the globe [3–5]. Plastic debris greatly varies in size and shape, but smaller particles (ex. < 5 mm) numerically dominate [6–8]. Once in aquatic systems, these materials are rapidly colonized by a diverse community of macro- and microorganisms, often referred to as the “Plastisphere” [9–12].

Surface-attached assemblies, as opposed to free living cells, benefit from the facilitated access to resources, enhanced interactions, and more stable environments that biofilms provide [13]. Consequently, Plastisphere microbiomes are distinct from planktonic communities [11, 14, 15], and typical genetic traits from biofilms are found, such as those involved in surface attachment [16]. Within a core group of bacterial families typically found in the Plastisphere (e.g., Flavobacteriaceae, Hyphomonadaceae, and Rhodobacteraceae) [11, 16, 17], bacterial communities mainly vary with season and geography [18–20]. Subtle colonization differences between polymer types have been shown [20, 21] although it remains unclear if these come as a consequence of the material’s polymer chemistry or its surface properties [15]. While the surrounding environment seems to be the main driver in shaping the general Plastisphere community [14, 20], species-specific variations between different materials draw interest as they may indicate target bacterial strains for biodegradation.

Despite encouraging findings in biodegradation of the polyester poly(ethylene terephthalate) (PET) [22, 23], biodegradation of non-hydrolyzable polymers, such as polyethylene (PE), is less likely to be encountered due to the high redox potential required to cleave the carbon-carbon bonds [24]. Nonetheless, similar molecules of lower molecular weight, i.e., n-alkanes, are commonly produced in marine environments [25], possibly feeding obligate hydrocarbon-degrading bacteria (OHCB) [26]. The latter can metabolize n-alkanes of up to ~ 50 carbons in length [27], which are notably shorter than the chains found in low density PE (C_4,000_–C_40,000_) and hence, abiotic weathering and reduction of polymer chain length is thought to be required to facilitate microbial biodegradation on non-hydrolysable plastics [28, 29]. Abiotic degradation can occur through photo- and thermal oxidation, adding functional groups to the polymer, such as carbonyl and hydroxyl groups, ultimately inducing chain scissions [30, 31]. Laboratory studies employing oxidized PE indeed demonstrated that weathered polymers lead to increased respiration rates [32], polymer weight loss [33–35], or stimulated microbial activity [36], but pre-weathered polymers were only recently considered in an in situ study of microbial plastic colonization [37]. Furthermore, pre-weathered polymers may mimic marine plastic debris as it occurs in the environment [38], and therefore the influence of weathered polymer surfaces on plastic colonization merits closer investigation, especially in light of the discovery of microorganisms potentially involved in polymer biodegradation.

Here, we tested the hypothesis that weathering a non-hydrolysable polymer (i.e., PE) enhances the colonization of OHCB taxa in the Plastisphere, while the untreated polymer and control material (i.e., glass) recruit more similar microbial communities with a lower relative abundance of OHCB. While our hypothesis held true during short incubations (i.e. weathered PE enriched a distinct group of microorganisms after two-day incubations), after 9 days the differences were no longer discernible between the materials and the relative abundance of these distinct microbes was drastically reduced. Our results suggest that more mature biofilms that develop on marine plastic debris mask polymer- or surface-specific microorganisms, hindering the detection of possible polymer biodegraders. Hence, mature biofilms likely consume labile organic matter generated from photosynthesis or from the surrounding environment—more than from the recalcitrant plastic itself.

## Materials and Methods

### Plastic Weathering and Monitoring of Surface Oxidation

Low density PE strips were obtained by heat pressing LDPE pellets (Sigma-Aldrich) into films (145 °C, 10 kN, pressing time 60 s, final thickness ~ 0.1 mm). The films were then cut into 0.5 × 1 cm strips and weathered by thermo-oxidation for 3 months at 80 °C in the dark; non-weathered PE strips were kept at room temperature. The carbonyl index (CI) was used as a measure of PE oxidation as done previously using Fourier-transform infrared spectroscopy (FTIR) [38, 39]. Briefly, the CI was calculated as the ratio between the carbonyl absorbance peak (1712 cm^−1^) and a standard PE reference that remains unaffected by weathering (2030 cm^−1^) [40]. FTIR spectra of PE were obtained in transmission mode by averaging 32 scans in the range of 600 to 4000 waves cm^−1^ with a resolution of 4 cm^−1^ (Spectrum GX, PerkinElmer). The CIs were measured for both weathered and non-weathered PE strips before in situ incubation and post incubation, after DNA had been extracted. An additional control was included to assess the effect of the DNA extraction process on the CI of weathered PE strips (but not exposed in situ). As additional control material, glass strips (~ 0.5 × 1 cm) were generated from microscope coverslips. Prior to experimental exposure, all strips were stored in absolute ethanol at room temperature.

### Experimental Setup and Sample Collection

In situ incubations in coastal seawater were performed in Mallorca (Spain, 39° 29′ 29.7″ N, 2° 44′ 09.0″ E) in August 2018 to study the marine microbial colonization of three materials: weathered and non-weathered PE strips, as well as the glass control. Twelve strips per material (*n*_total_ = 36) were fixed to nylon fishing lines with electrical tape, attaching each end of the line to a weight and buoy, which maintained the strips at ~ 1.5 m depth in a ~ 3 m-deep coastal rocky region. Six strips of each material were recovered at each one of the two time points (i.e., 2 and 9 days) and immediately immersed in 1-mL lysis buffer (Qiagen) and stored at − 20 °C until further analysis. Additionally, the surrounding planktonic community was sampled at day 9 by filtering in situ 2.5 L of seawater through a 0.2-μm filter membrane (GTTP, Isopore, Millipore). Seawater samples were collected in triplicate and filters were immediately stored in 1 mL of lysis buffer at − 20 °C.

### Primer Pair Coverage of OHCB

Given the particular interest to study OHCB among the communities, the universal 16S rRNA gene primer pair employed here (Supplementary Table S1) was assessed for its coverage of a subset of important taxa of the OHCB group: *Alcanivorax*, *Oleiphilus*, *Oleispira*, *Thalassolituus*, *Cycloclasticus*, *Marinobacter*, *Neptunomonas*, and *Thalassospira* [41]. For comparison, the general primers used in recent Plastisphere surveys were also tested for their coverage of OHCB group [11, 17, 42], as well as the primer pair suggested by Berry and Gutierrez [41], due to best coverage for OHCB among general primer sets (Supplementary Table S1). All primer pairs were assessed with the database SILVA SSU 132 Ref NR. In silico testing was performed with TestPrime [43] v1.0 on the ARB PT server using the most conservative setting (“0 mismatches”).

### DNA Isolation, Amplification, and Library Generation

DNA from the biofilms of PE and glass, as well as seawater communities, was extracted using the DNeasy PowerBiofilm kit (Qiagen) according to the manufacturer’s instructions, which included a bead-beating step. DNA was quantified using a Qubit® HS DNA kit (Life Technologies Corporation) and samples were diluted to equalize the concentration. PCR amplifications were performed using Q5® Hot Start High-Fidelity 2X Master Mix (New England Biolabs® inc.) and the primer pair 515F-Y and 926R ([44, 45], Supplementary Table S1), which amplified regions V4-5 of the 16S rRNA gene of bacteria, using PCR conditions as described previously [45]. PCR products were purified with Ampliclean magnetic beads (Nimagen, The Netherlands). Index PCR was performed using Illumina Nextera Index Kit v2 adapters. Sample normalization was done with the SequelPrep™ Normalisation Plate Kit (ThermoFisher Scientific) and samples were pooled for sequencing. Pooled libraries were quantified using the NEBNext Library Quant Kit for Illumina (New England Biolabs, UK) and diluted to 4 nM. Negative DNA extraction controls and negative controls for sequencing were processed simultaneously.

### 16S rRNA Gene Amplicon Sequencing and Processing

Libraries were denatured using 0.2 N NaOH and sequenced using the MiSeq Illumina system (2 × 300 bp paired-end) with the v3 reagent kit, following the manufacturer’s instructions for a 14 pM library with 2% phiX as an internal reference. Sequence processing was performed in R v3.5.1 [46], where amplicon sequence variants (ASVs) [47] were obtained using the DADA2 package [48]. Forward and reverse primer sequences as well as fragment ends with low quality scores were trimmed, yielding final lengths of 276 bp and 200 bp for forward and reverse reads, respectively. Chimeras were removed and taxonomy was then assigned using IDTAXA [49] implemented in the R package DECIPHER [50] with a classifier trained on the SILVA v132 database (March 2018 release). A maximum likelihood phylogenetic tree was then built using the GTRGAMMA model in RAxML [51]. All raw sequence files, including sequencing controls, are available from the NCBI Short Read Archive (SRA) database (BioProject PRJNA528407).

### Data Analysis and Statistics

Prior to downstream analysis, unassigned reads at the phylum level were removed due to high likelihood of representing artefacts. 16S rRNA gene sequences assigned to chloroplasts and mitochondria were removed, as well as phyla with < 9 reads across all samples. Data from all samples, including controls, were first inspected via principal coordinate analysis (Bray-Curtis distance). Ensuing, samples with < 1000 reads, as well as outliers and controls (i.e., extraction and sequencing blanks), were removed from the data, and taxa were then agglomerated at the genus level without removing non-assigned features. Sequencing coverage was inspected via rarefying curves. To investigate the α-diversity of the communities, indices were calculated for Shannon diversity, inverted Simpson evenness, and Chao 1 richness. Differences in Shannon diversity were further assessed for their statistical difference via generalized linear modelling using a Gamma link function followed by all pairwise comparisons; the Shannon index was chosen because it is less sensitive to differences between library sizes than other indices [52]. Then, β-diversity was investigated through non-metric multidimensional scaling (nMDS) using the UniFrac distance metric, both weighted and unweighted [53]. For weighted UniFrac, proportion transformed data were used, while unweighted UniFrac was performed on rarefied data in accordance with Weiss et al. [54]. Permutation tests (Adonis [55]) were used to statistically explore differences in β-diversity between communities in response to experimental treatments using the UniFrac distance metrics (weighted and unweighted). To find taxa of interest, differential abundance testing was performed via the DESeq2 package in R [56] using raw counts; DESeq2 employs negative binomial generalized linear models, controls for different library sizes and corrects for multiple testing with the Benjamini-Hochberg procedure. The closest cultivated relatives of the taxa of interest were identified through BLAST searches on the National Center for Biotechnology Information (NCBI) against the 16S rRNA gene sequence database. The 16S rRNA gene sequences of distinct ASVs used in BLAST searches are provided as supplementary information.

Data analysis, statistics, and plotting in R further included the following packages: phyloseq [57], multcomp [58], and ggplot2 [59].

## Results

### Weathering of the PE Strips

**Fig. 1:**
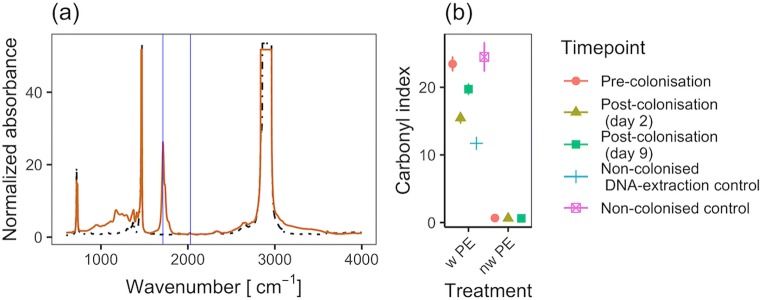
Polyethylene (PE) weathering. **a** Representative FTIR spectra of weathered (orange line) and non-weathered PE (dot-dashed black line). The peaks used for calculating the carbonyl index (CI) are indicated (blue vertical lines): carbonyl peak at 1712 cm^−1^ and internal reference at 2030 cm^−1^; **b** CI (± standard error, *n* = 3) obtained from weathered PE (w PE), and non-weathered PE (nw PE) after different experimental exposures

FTIR spectra confirmed thermal oxidation of the PE strips that had been kept at 80 °C for 3 months (CI = 23.5; Fig. 1), comparable with 270 days of UV exposure at 43–45 °C [39], but higher than what has been measured from marine plastic debris, i.e., CI < 1 [38, 60]. Interestingly, CIs decreased after PE strips had been incubated in seawater (CIs of 15.5 and 19.7 after 2 and 9 days of in situ incubations, respectively; Fig. 1b). Control strips that only went through the DNA extraction protocol also showed a reduction in their CI (CI = 11.7), whereas surface oxidation remained stable in weathered PE strips that were not processed and remained at room temperature for the duration of the experiment (not stored in absolute ethanol). These results indicated that oxidized polymer chains from the surface of weathered plastics shed off when the material was in solution as suggested previously [36, 61].

### Primer Pair Coverage of OHCB

In silico analysis of different 16S rRNA gene primer pairs showed that those used in this study covered 92% of the OHCB present in the reference SILVA database (*n* = 1867, Table 1). Similar coverage was obtained by other primer pairs (i.e., 91%) used in recent Plastisphere surveys [17, 42], and was not far from the coverage obtained with an ideal general primer pair (i.e., 93%) suggested by Berry and Gutierrez [41]. In agreement with this last study [41], we found that the primer pair 518F and 1046R only covered 36% of the OHCB, mainly due to poor coverage of the *Marinobacter* genus (3%, Table 1). Primer pair 515F-Y and 926R was used in the present study because it gave both a good coverage of the OHCB group, as well as the best coverage for marine microbial communities as previously suggested [45].

**Table 1:** Coverage of important OHCB genera obtained by different universal 16S rRNA primer pairs

Primer^a^		HVR^b^	Sequences by genus^c^								Total target sequences	Representative plastisphere study
Forward	Reverse		Alcanivorax	Oleiphilus	Oleispira	Thalassolituus	Cycloclasticus	Marinobacter	Neptunomonas	Thalassospira		
343F	908R	V3-5	0.87	0.98	0.96	0.90	0.95	0.94	0.97	0.95	**0.93**	Berry & Gutierrez 2017 [41]
515F-Y	926R	V4-5	0.88	0.98	0.93	0.90	0.90	0.93	0.95	0.90	**0.92**	this study
515F	806R	V4	0.88	0.91	0.89	0.95	0.92	0.92	0.92	0.90	**0.91**	Oberbeckmann et al. 2018 [42]
341F	785R	V3-4	0.86	0.93	0.89	0.95	0.97	0.92	0.97	0.93	**0.91**	Kirstein et al. 2018 [21]
341F	805R	V3-4	0.86	0.93	0.89	0.95	0.97	0.92	0.97	0.94	**0.91**	De Tender et al. 2017 [17]
518F	1046R	V4-6	0.76	1.00	0.93	0.85	0.92	0.03	0.95	0.90	**0.36**	Zettler et al. 2013 [11]

^a^For primer sequences and details see Supplementary Table S1

^b^HVR: hyper variable region of 16S rRNA genes

^c^Total target sequences: *n* = 1867; Alcanivorax sequences: *n* = 397; Oleiphilus sequences: *n* = 45; Oleispira sequences: *n* = 27; Thalassolituus sequences: *n* = 20; Cycloclasticus sequences: *n* = 59; Marinobacter sequences: *n* = 1104; Neptunomona sequences: *n* = 38; Thalassospira sequences: *n* = 177

### Analysis of the Plastispheres

16S rRNA gene sequencing data were obtained from biofilms that colonized weathered and non-weathered PE as well as glass strips after 2 and 9 days of incubation in coastal marine water of the Mediterranean sea (*n* = 6 for each material and time point). These were inspected together with the 16S rRNA gene data from the planktonic community of the surrounding seawater (*n* = 3, day 9) and controls: extraction kit blank (*n* = 2) and negative PCR amplifications (n = 2). Plastisphere communities were distinct from the planktonic seawater community, as well as controls, except for some samples that were identified as outliers and discarded from downstream analysis, as they had < 1000 reads (similar to blanks) or clustered with blank extraction controls (i.e., 3× non-weathered PE from day 9, 1× non-weathered PE from day 2, and 1× glass from day 2; Supplementary Fig. S1). The mean number of reads for the samples from day 9 (9492 ± 1543 SE) was lower than that obtained from day 2 (25,081 ± 3066 SE), impacting the coverage of ASV richness (Supplementary Fig. S2). The agglomerated dataset contained 495 taxa in 22 phyla, of which Proteobacteria (*n* = 243 taxa and 59.9% overall relative abundance) and Bacteroidetes (*n* = 89 taxa and 28.9% overall relative abundance) were best represented (Supplementary Figs. S3 and S4).

**Fig. 2:**
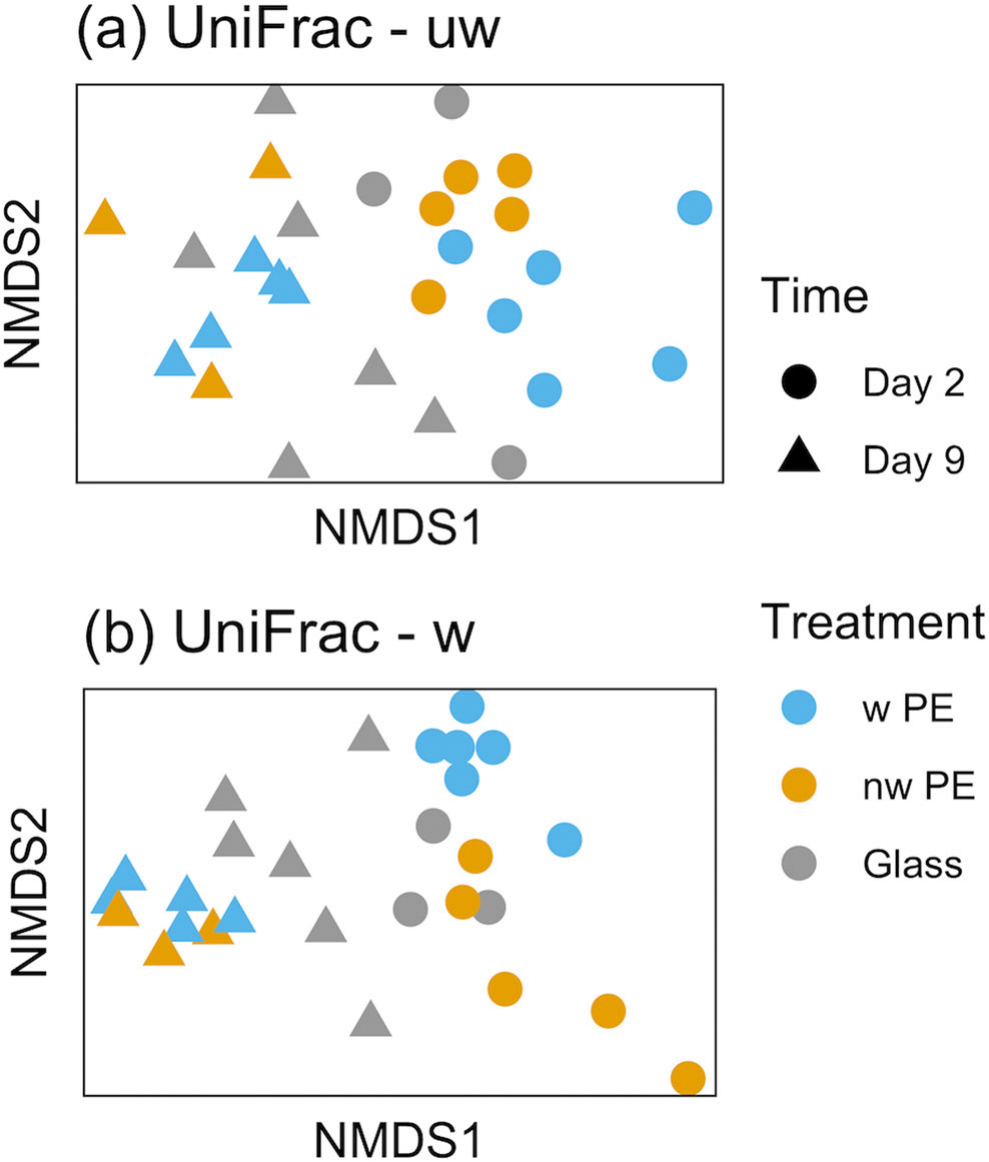
Nonmetric multidimensional scaling (nMDS) plots of bacterial communities (16S rRNA gene) colonizing weathered PE (w PE), non-weathered PE (nw PE) and glass in coastal Mediterranean seawater after 2 and 9 days of incubation. Ordinations based on UniFrac distances, both unweighted **a** and weighted **b**. **a***k* = 2 axes, stress 0.224; **b***k* = 2 axes, stress 0.072

**Fig. 3:**
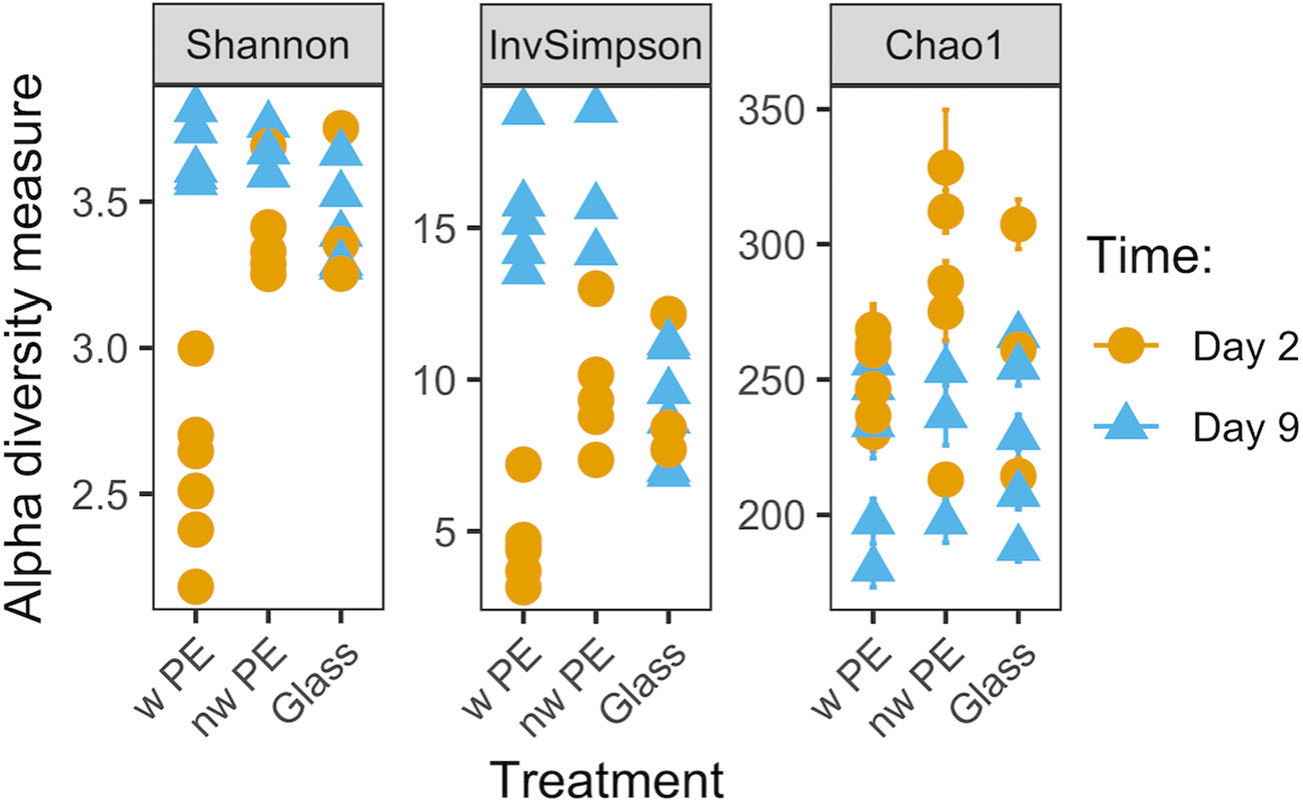
Alpha diversity measures of bacterial communities (16S rRNA gene) on weathered polyethylene (w PE), non-weathered PE (nw PE), and glass after 2 and 9 days of incubation in coastal Mediterranean seawater

While the microbial communities differed between the two time points (*p* = 0.001 for both weighted and unweighted UniFrac; statistical summary is in Supplementary Table S2), they did not differ as consistently between the three different materials (unweighted UniFrac *p* = 0.077; weighted UniFrac *p* = 0.001; Supplementary Table S2), and stress values suggested that the weighted UniFrac fit the data better on 2 axes (0.072 for weighted vs. 0.224 for unweighted UniFrac, Fig. 2). This indicates that all materials were colonized by similar organisms (less support for the measure of presence-absence, i.e., unweighted UniFrac; Fig. 2a), but their abundance differed between materials which drove differentiation as indicated by the weighted UniFrac analysis (Fig. 2b). Nonetheless, after longer incubations (i.e., 9 days), this difference between materials was lost and all communities converged (Fig. 2). The α-diversity measures confirmed this pattern demonstrating that the communities on weathered PE at day 2 were the least diverse (Shannon index in Fig. 3; see Supplementary Table S3 for statistical summary), and also least even (InvSimpson, Fig. 3), while ASV richness showed greater overlap with other treatment combinations (Chao1, Fig. 3). Shannon diversity remained similar for all other treatment combinations (Fig. 3; Supplementary Table S3).

### Distinctness of the Plastisphere on Weathered PE

**Fig. 4:**
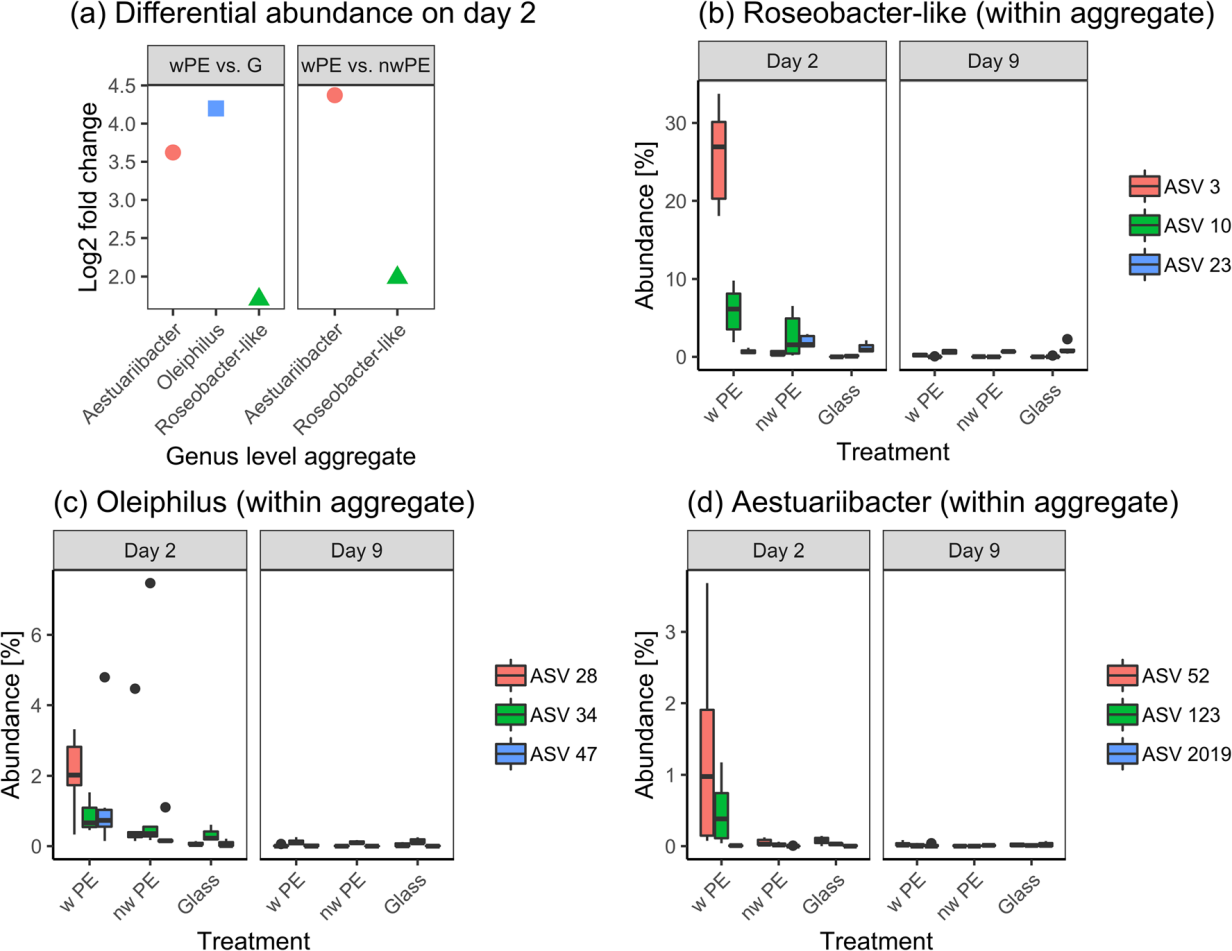
Differentially abundant amplicon sequence variants (ASVs) from polyethylene (PE; w: weathered; nw: non-weathered) and glass (G). **a** Log2 fold changes for differentially abundant ASVs (aggregated at genus level). Alteromonadales (red circles), Rhodobacterales (green triangles) and Oceanospirillales (blue square) are indicated. **b–d** The three most abundant ASVs within each aggregated genus are shown in boxplots displaying median relative abundance using DESeq2 normalized counts. **b***Roseobacter*-like (*n*_tot_ = 2119 ASVs), **c***Oleiphilus* (*n*_tot_ = 28 ASVs), **d***Aestuariibacter* (*n*_tot_ = 24 ASVs). Details of the ASVs can be found in Supplementary Table S4

Here, we confirm that the low α-diversity displayed by the Plastisphere communities of weathered PE at day 2 (observed in Figs. 2b and 3) was due to a small number of abundant species that drove the differentiation of the community. The aggregate of ASVs assigned to the family Rhodobacteraceae constituted 7488 DESeq2 normalized counts (43.5% of the community) on weathered PE, which was 4× higher than non-weathered PE (normalized counts = 1900, log2 fold change = 2, *p* = 3.4 × 10^−10^; Fig. 4a). Interestingly, inspection of the individual ASVs that had been aggregated within a genus belonging to the Rhodobacteraceae revealed that this difference between materials was mainly driven by a single ASV (ASV3). ASV3 represented 27% median of the prokaryotic community on weathered PE at day 2 while its relative abundance remained below 0.4% on both non-weathered PE and glass (Fig. 4b). At day 9 though, the median relative abundance of ASV3 on weathered PE had dropped to 0.23% (Fig. 4b). A BLAST search of ASV3 returned *Thalassococcus halodurans* as the closest hit (99.73% 16S rRNA gene sequence identity), but taxonomic assignment remained inconclusive due to other close matches and we therefore refer to this ASV as a *Roseobacter*-like organism.

The genera *Oleiphilus* (Order: Oceanospirillales and representative of the OHCB group) and *Aestuariibacter* (Order: Alteromonadales) represented ~ 5.8% and ~ 1.8% of the prokaryotic community on weathered PE on day 2, respectively (Fig. 4c, d). While both organisms were overrepresented in communities from weathered PE compared with Glass (*Oleiphilus*, log2 fold change = 4.2, *p* = 1.4 × 10^−5^; *Aestuariibacter*, log2 fold change = 3.6, *p* = 9 × 10^−4^; Fig. 4a), only *Aestuariibacter* remained differentially abundant when comparing weathered PE to non-weathered PE (log2 fold change = 4.4, *p* = 3.2 × 10^−8^; Fig. 4a). The most abundant amplicon sequence variant belonging to *Oleiphilus* (ASV28) showed 94.4% 16S rRNA gene sequence identity with *Oleiphilus messinensis*. The abundant *Aestuariibacter* ASVs, i.e., ASV52 and ASV123, displayed 97.3% sequence identity to *Aestuariibacter aggregatus*.

## Discussion

We show that weathered PE surfaces incubated in coastal seawater initially selected for a less diverse microbial community compared with both untreated PE and glass, mainly due to the short-term enrichment of several distinct bacteria. Identifying bacterial communities on marine plastic debris has been the main focus of a number of recent environmental surveys [14, 17, 18, 20, 42]. These studies revealed that geographical and seasonal factors were better predictors of Plastisphere community differentiation than the actual polymer type itself. We believe that the reason for such observations is that these analyses are usually done on mature Plastispheres that have spent weeks, if not months, at sea allowing communities to develop and converge.

Here, for the first time, we analyzed the very early colonization of weathered PE in comparison with non-weathered PE, as well as glass, and observed the enrichment of mainly three organisms: *Roseobacter-*, *Oleiphilus-*, and *Aestuariibacter*-like taxa. The *Roseobacter* group*-*like organism was particularly abundant only on weathered PE (ASV3; 27% of the prokaryotic community and 90× more abundant than on the other two materials). Despite the fact that *Rhodobacteraceae*, and especially taxa from the *Roseobacter* group, are known primary colonizers of surfaces in marine environments [62, 63], the specificity of ASV3 for weathered PE is notable. Members of the *Roseobacter* group are known for their high versatility, and in some cases, their ability to degrade certain hydrocarbon compounds [64–66], though further experimentation is required to confirm that this enriched *Roseobacter*-like strain is able to metabolize subproducts released from the weathered material.

The other two enriched genera on weathered PE, i.e., *Oleiphilus* and *Aestuariibacter*, are known hydrocarbon degraders [67, 68]. *Oleiphilus* is a member of the OHCB group, and is “specialized” in degrading n-alkanes in the C_11_–C_20_ range [67, 69], which would be consistent with the molecules generated by PE weathering [29, 61]. While OHCB are generally reported within the rare taxa of the Plastisphere, here, we observed a considerable relative abundance of *Oleiphilus* during the early colonization stages of PE (i.e., 5.8 and 3.7% on weathered and non-weathered PE, respectively). Unlike *Oleiphilus*, *Aestuariibacter* was preferentially enriched only on weathered PE (1.8% relative abundance and almost 38× more abundant than on non-weathered PE). In this context, *Aestuariibacter* and *Oleiphilus* are interesting organisms that deserve further attention.

Recalcitrant polymers used to manufacture plastic materials, e.g., PE, are highly inert and difficult to biodegrade [24]. Earlier laboratory studies described the release of short-chain compounds from weathered plastics which ultimately enhanced microbial growth [29, 36, 61, 70]. The abiotic reduction in molecular weight of synthetic polymers may be crucial prior to any potential biodegradation [28], as chain scission products such as suberic- or tetradecanedioic acid from photooxidized PE [61] are more amenable for bacterial growth. In light of current marine plastic waste issues, interest in biodegradation of common non-hydrolysable polymers has soared, but the importance of plastic oxidation has only recently been considered for in situ colonization studies, confirming tangible treatment effects on plastic colonization [37, 71].

We believe that the higher load of chain scission products from the weathered PE enriched, rather than selected, for the distinct genera given that the weighted-, but not the unweighted, UniFrac-based analyses supported community differentiation, thus indicating that the observed differences were due to relative abundance of community members, instead of the presence-absence of taxa. Crucially, it remains unknown whether such strains merely scavenge released chain scission products (indirect degradation), or if the microorganisms are capable of direct degradation of high molecular weight polymers such as PE, given that the latter requires potent extracellular oxidizing enzymes [24].

Molecular characterization and further confirmation of plastic biodegraders can only be achieved by isolation. Nonetheless, we show that isolation efforts of putative biodegrading microorganisms should target very early stages of plastic colonization as more mature biofilms on PE and glass converged, the relative abundance of initially enriched genera decreased, and differences at the community level were no longer evident. Similarly, in an experiment over 45 days, Dussud et al. [37] reported a 1.7–3 fold higher relative abundance of (O)HCB on all tested polymer types in early colonization stages, when compared with seawater communities. Moreover, these observations are in accordance with chitin particle colonization experiments, which showed how early colonization of biodegrading organisms were later replaced by non-degrading secondary consumers [72, 73]. Later biofilm stages may thus complicate the identification and isolation of microbial candidates for further study of microbial biodegradation of plastics. Alternatively, the secondary biofilm could be removed to reveal the rare and tightly attached organisms on the surface of plastics, as recently reported [74].

The present study meets a research gap in the context of biodegradation, highlighting that the isolation of potentially interesting taxa should involve sampling at earlier stages of surface colonization and using pre-oxidized polymers. While recalcitrant plastics do not appear to serve as an important carbon source for mature Plastispheres, early colonizing organisms display potential to metabolize subproducts emerging from plastic weathering. Whether these microbes are able to carry out the first steps of surface oxidation remains an open question.

## Electronic Supplementary Material

ESM 1 (PDF 1185 kb)
