# Supplementary material for: Early Colonization of Weathered Polyethylene by Distinct Bacteria in Marine Coastal Seawater
Source: Microb Ecol. 2019 Aug 29;79(3):517–26. doi: 10.1007/s00248-019-01424-5 (PMC7176602; doi:10.1007/s00248-019-01424-5)
Supplement: Supplementary file 1 — (PDF 1185 kb) [file 248_2019_1424_MOESM1_ESM.pdf]

SUPPLEMENTARY MATERIAL

Early colonization of weathered polyethylene by distinct bacteria in marine coastal seawater

Gabriel Erni-Cassola,[1*] Robyn J. Wright,[1] Matthew I. Gibson,[2,3] Joseph A. Christie-Oleza[1*]

[1] School of Life Sciences, University of Warwick, Coventry CV4 7AL, UK.

[2] Department of Chemistry, University of Warwick, Coventry CV4 7AL, UK.

[3] Warwick Medical School, University of Warwick, Coventry CV4 7AL, UK.

*corresponding authors at: School of Life Sciences, University of Warwick, Coventry CV4 7AL, UK.

g.ernicassola@warwick.ac.uk/gabriel.ernicassola@gmail.com and J.Christie-Oleza@warwick.ac.uk

This file contains 11 pages (cover page included), containing 4 figures, 4 tables and on page S9 specific 16S rRNA gene sequences used in BLAST searches:

Table S1: 16S rRNA gene primer pairs tested for OHCB coverage.

Figure S1: Principal coordinate analysis (PCoA; Bray-Curtis distance) plot of the full 16S rRNA gene sequencing data.

Figure S2: Rarefaction curves for bacterial communities

Figure S3: Bar chart showing dominant bacterial phyla

Figure S4: Bar chart showing dominant bacterial orders

Table S2: Statistical summary of PERMANOVA tests on UniFrac ordinations of 16S rRNA data from polyethylene communities.

Table S3: Contrast summaries of generalized linear model results for Shannon diversity of bacterial communities (16S rRNA gene) colonizing PE and glass at two different time points.

Table S4: Details for most abundant ASVs from figure 4.

Table S1. 16S rRNA gene primer pairs tested for OHCB coverage.

Primer name	Sequence	Reference
515F-Y(*)	GTGYCAGCMGCCGCGGTAA	Parada *et al.* [1]
926R(*)	CCGYCAATTYMTTTRAGTTT	Quince *et al.* [2]
515F	GTGCCAGCMGCCGCGGTAA	Oberbeckmann *et al.* [3]
806R	GGACTACHVGGGTWTCTAAT	
341F	CCTACGGGNGGCWGCAG	De Tender *et al.* [4]
805R	GACTACHVGGGTATCTAATCC	
518F	CCAGCAGCYGCGGTAAN	Zettler *et al.* [5]
1046R	CGACAGCCATGCANCACCT	
341F	CCTACGGGNGGCWGCAG	Kirstein *et al.* [6]
785R	GACTACHVGGGTATCTAATCC	
343F	TACGGRAGGCAGCAG	Berry and Gutierrez [7]
908R	CGTCAATTCMTTTGAGTT	

Note: (*) indicates primer pair used in this study.

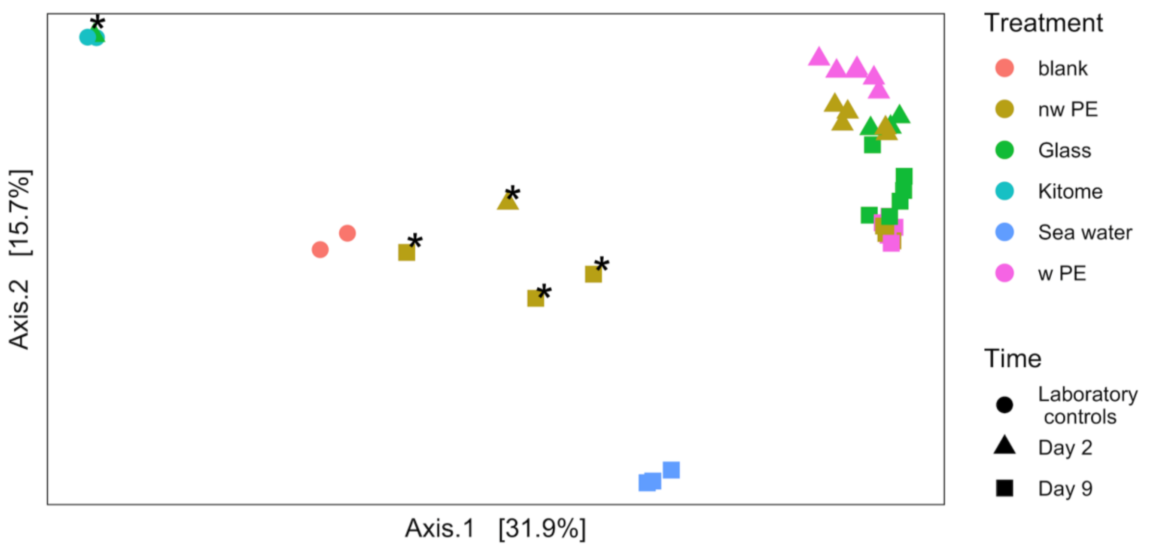

Figure S1. Principal coordinate analysis (PCoA; Bray-Curtis distance) plot of the full 16S rRNA gene sequencing data. Note that controls (Kitome, blank), as well as Sea water samples cluster separately from bulk experimental samples (nw PE, w PE and Glass). (*): experimental samples that were excluded from downstream analysis due to clustering with controls.

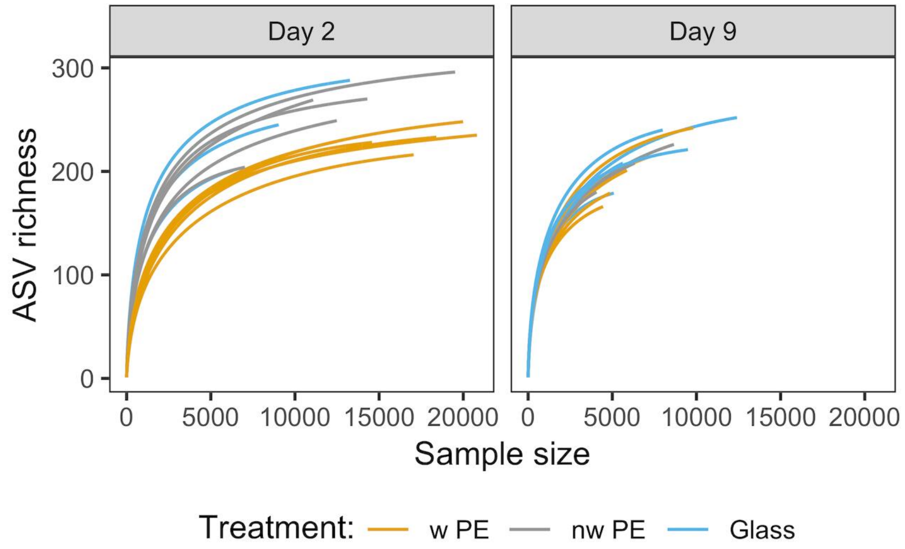

Figure S2. Rarefaction curves for bacterial communities (16S rRNA genes) colonizing weathered polyethylene (w PE), non-weathered PE (nw PE), and glass after 2 and 9 days of incubation in coastal Mediterranean seawater.

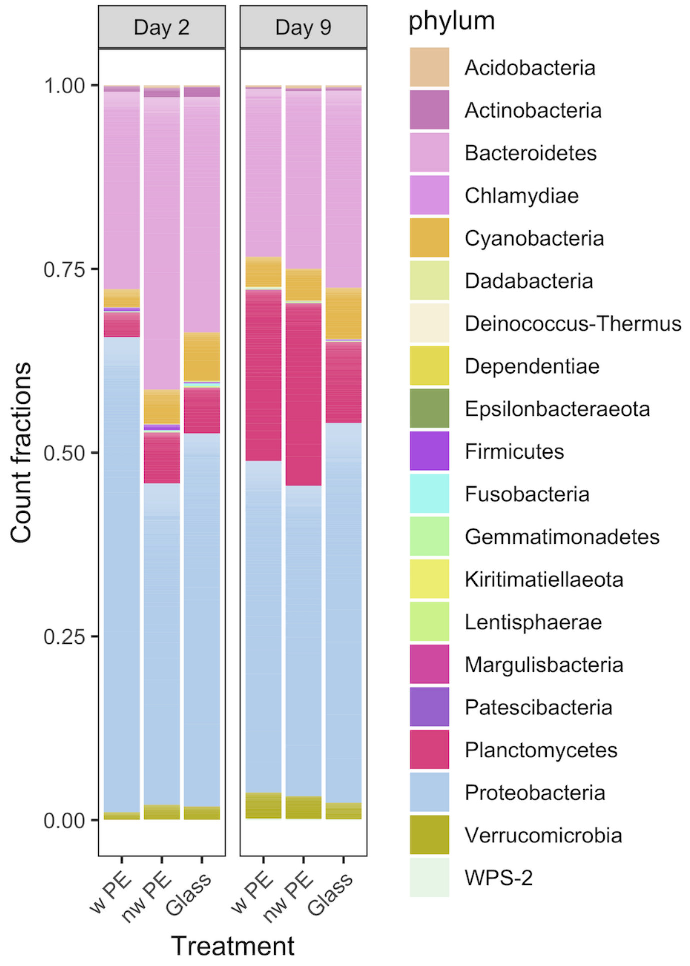

Figure S3. Bar chart showing dominant bacterial phyla (16S rRNA genes) in microbial communities colonizing weathered polyethylene (w PE), non-weathered PE (nw PE), and glass after 2 and 9 days of incubation in coastal Mediterranean seawater.

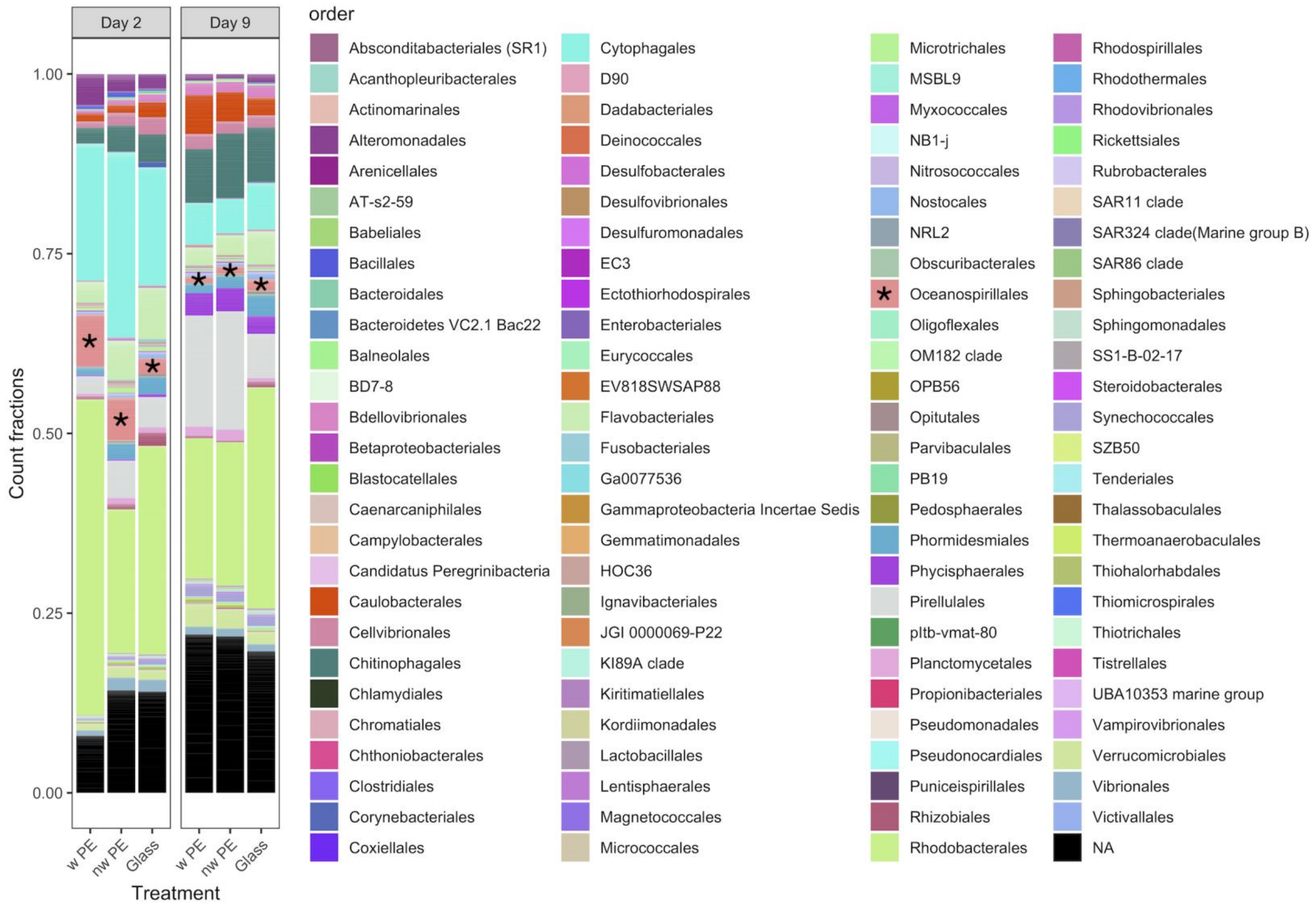

Figure S4. Bar chart showing dominant bacterial orders (16S rRNA gene) in microbial communities colonizing weathered polyethylene (w PE), non-weathered PE (nw PE), and glass after 2 and 9 days of incubation in coastal Mediterranean seawater. (*) denotes genus *Oceanospirillales*

Table S2. Statistical summary of PERMANOVA tests on UniFrac ordinations of 16S rRNA gene data from polyethylene communities.

			16S rRNA gene			
**UniFrac: weighted**	**Df**	**Sums of Sqs**	**Mean Sqs**	**F Model**	**R^2**	**Pr(>F)**
treatment	2	1.450	0.725	5.916	0.147	**0.001**
timepoint	1	4.588	4.588	37.453	0.466	**0.001**
treatment:timepoint	2	1.108	0.554	4.522	0.113	**0.003**
Residuals	22	2.695	0.123		0.274	
**UniFrac: unweighted**						
treatment	2	0.153	0.077	1.304	0.084	0.077
timepoint	1	0.239	0.239	4.074	0.131	**0.001**
treatment:timepoint	2	0.147	0.074	1.255	0.080	0.098
Residuals	22	1.291	0.059		0.705	

Note: significant p-values are denoted in bold.

Table S3. Contrast summaries of generalized linear model results for Shannon diversity of bacterial communities (16S rRNA gene) colonizing PE and glass at two different time points.

Compared conditions	Estimate	Std. Error	z value	Pr(>|z|)
glass, day 2 – non-weathered PE, day 2	-0.005	0.014	-0.354	0.999
**weathered PE, day 2 – non-weathered PE, day 2**	0.094	0.014	6.861	**<0.001**
non-weathered PE, day 9 – non-weathered PE, day 2	-0.023	0.014	-1.649	0.563
glass, day 9 – non-weathered PE, day 2	-0.001	0.012	-0.089	1.000
weathered PE, day 9 – non-weathered PE, day 2	-0.022	0.012	-1.811	0.455
**weathered PE, day 2 – glass, day 2**	0.100	0.015	6.467	**<0.001**
non-weathered PE, day 9 – glass, day 2	-0.018	0.015	-1.145	0.861
glass, day 9 – glass, day 2	0.004	0.014	0.289	1.000
weathered PE, day 9 – glass, day 2	-0.017	0.014	-1.207	0.832
**non-weathered PE, day 9 – weathered PE, day 2**	-0.117	0.015	-7.852	**<0.001**
**glass, day 9 – weathered PE, day 2**	-0.096	0.013	-7.196	**<0.001**
**weathered PE, day 9 – weathered PE, day 2**	-0.116	0.013	-8.692	**<0.001**
glass, day 9 – non-weathered PE, day 9	0.022	0.013	1.631	0.575
weathered PE, day 9 – non-weathered PE, day 9	0.001	0.013	0.064	1.000
weathered PE, day 9 –glass, day 9	-0.021	0.011	-1.809	0.457

Note: significant p-values are denoted in bold.

Table S4. Details for most abundant ASVs from figure 4.

ASV	Abundance on wPE at day2 [%]	Closest relative	Identity score
ASV3 [1]	26.9	*Thalassococcus halodurans*	99.73%
ASV10 [2]	6.1	*Marivivens niveibacter*	98.66%
ASV23 [3]	0.6	*Boseongicola aestuarii*	97.31%
ASV28 [4]	2.1	*Oleiphilus mesinensis*	94.41%
ASV34 [5]	0.8	*Oleiphilus mesinensis*	95.48%
ASV47 [6]	1.3	*Oceanospirillum nioense*	94.39%
ASV52 [7]	0.9	*Aestuariibacter aggregatus*	97.33%
ASV123 [8]	0.4	*Aestuariibacter aggregatus*	97.33%
ASV2019 [9]	0.01	*Aestuariibacter aggregatus*	98.40%

[1]

TACGGAGGGGGTTAGCGTTGTTCGGAATTACTGGGCGTAAAGCGCGCGTAGGCGGATTGGAAAGTTGGGGGTGAAATCCCGGGGCTCAACCCCGGAAC
TGCCTCCAAAACTATCAGTCTAGAGTTCGAGAGAGGTGAGTGGAATTCCGAGTGTAGAGGTGAAATTCGTAGATATTCGGAGGAACACCAGTGGCGAA
GGCGGCTCACTGGCTCGATACTGACGCTGAGGTGCGAAAGTGTGGGGAGCAAACAGGATTAGATACCCTGGTAGTCCACACCGTAAACGATGAATGCC
AGTCGTCAGCAAGCATGCTTGTTGGTGACACACCTAACGGATTAAGCATTCCGCCTGGGGAGTACGGTCGCAAGATTA

[2]

TACGGAGGGGGTTAGCGTTGTTCGGAATTACTGGGCGTAAAGCGCACGTAGGCGGACTGGAAAGTTAGAGGTGAAATCCCGGGGCTCAACCCCGGAAC
TGCCTTTAAAACTATCAGTCTAGAGTTCGAGAGAGGTGAGTGGAATTCCGAGTGTAGAGGTGAAATTCGTAGATATTCGGAGGAACACCAGTGGCGAA
GGCGGCTCACTGGCTCGATACTGACGCTGAGGTGCGAAAGTGTGGGGAGCAAACAGGATTAGATACCCTGGTAGTCCACACCGTAAACGATGAATGCC
AGTCGTCGGGTTGCTTGCAACTCGGTGACACACCTAACGGATTAAGCATTCCGCCTGGGGAGTACGGTCGCAAGATTA

[3]

TACGGAGGGGGTTAGCGTTGTTCGGAATTACTGGGCGTAAAGCGCACGTAGGCGGATTATTAAGTGAGAGGTGAAATCCCAGGGCTCAACCCTGGAAC
TGCCTTTCATACTGGTAATCTTGAGTTCGAGAGAGGTGAGTGGAATTCCAAGTGTAGAGGTGAAATTCGTAGATATTTGGAGGAACACCAGTGGCGAA
GGCGGCTCACTGGCTCGATACTGACGCTGAGGTGCGAAAGTGTGGGGAGCAAACAGGATTAGATACCCTGGTAGTCCACACCGTAAACGATGAATGCC
AGACGTCGGGCAGTATACTGTTCGGTGTCACACCTAACGGATTAAGCATTCCGCCTGGGGAGTACGGTCGCAAGATTA

[4]

TACGGAGGGTGCAAGCGTTAATCGGAATTACTGGGCGTAAAGCGCGCGTAGGCGGCTTACTAAGCCAGATGTGAAAGCCCCGGGCTCAACCTGGGAAC
TGCATTTGGAACTGGTTCGCTAGAGTACAGTAGAGGGTGGTGGAATTTCCAGTGTAGCGGTGAAATGCGTAGAGATTGGAAGGAACATCAGTGGCGAA
GGCGGCCACCTGGACTGATACTGACGCTGAGGTGCGAAAGCGTGGGGAGCAAACAGGATTAGATACCCTGGTAGTCCACGCCGTAAACGATGTCTACT
AGCCGTTGGGGATCTTGTATCTTTAGTGGCGCAGCTAACGCACTAAGTAGACCGCCTGGGGAGTACGGCCGCAAGGTTA

[5]

TACGGAGGGTGCAAGCGTTAATCGGAATTACTGGGCGTAAAGCGCGCGTAGGCGGTTGCTTAAGCCAGATGTGAAAGCCCCGGGCTCAACCTGGGAAC
TGCATTTGGAACTGGGCAGCTAGAGTACAGTAGAGGGTGGTGGAATTTCCAGTGTAGCGGTGAAATGCGTAGAGATTGGAAGGAACATCAGTGGCGAA
GGCGGCCACCTGGACTGATACTGACGCTGAGGTGCGAAAGCGTGGGGAGCAAACAGGATTAGATACCCTGGTAGTCCACGCCGTAAACGATGTCTACT
AGCCGTTGGGGAACTTGATTCTTTAGTGGCGCAGCTAACGCGATAAGTAGACCGCCTGGGGAGTACGGCCGCAAGGTTA

[6]

TACGGAGGGTGCAAGCGTTAATCGGAATTACTGGGCGTAAAGCGCGCGTAGGCGGCTTACTAAGCCAGATGTGAAAGCCCAGGGCTTAACCTTGGAAC
TGCATTTGGAACTGGTTCGCTAGAGTACAGTAGAGGGTGGTGGAATTTCCAGTGTAGCGGTGAAATGCGTAGAGATTGGAAGGAACATCAGTGGCGAA
GGCGGCCACCTGGACTGATACTGACGCTGAGGTGCGAAAGCGTGGGGAGCAAACAGGATTAGATACCCTGGTAGTCCACGCCGTAAACGATGTCTACT
AGCCGTTGGGGAACTTGATTCTTTAGTGGCGCAGCTAACGCACTAAGTAGACCGCCTGGGGAGTACGGTCGCAAGATTA

[7]

TACGGAGGGTGCGAGCGTTAATCGGAATTACTGGGCGTAAAGCGCACGCAGGCGGATTGTTAAGCTAGAGGTGAAAGCCCCGCGCTCAACGTGGGAAT
TGCCTTTAGAACTGGCAGTCTAGAGTCTTGGAGAGGGGAGTGGAATTCCAGGTGTAGCGGTGAAATGCGTAGAGATCTGGAGGAACATCAGTGGCGAA
GGCGACTCCCTGGCCAAAGACTGACGCTCATGTGCGAAAGTGTGGGTAGCGAACAGGATTAGATACCCTGGTAGTCCACACCGTAAACGCTGTCTACT
AGCTGTTTGTGGTTTTAAACCGTGAGTAGCGAAGCTAACGCGCTAAGTAGACCGCCTGGGGAGTACGGCCGCAAGGTTA

[8]

TACGGAGGGTGCGAGCGTTAATCGGAATTACTGGGCGTAAAGCGCACGCAGGCGGATTGTTAAGCTAGAGGTGAAAGCCCCGCGCTCAACGTGGGAAT
TGCCTTTAGAACTGGCAGTCTAGAGTCTTGGAGAGGGGAGTGGAATTCCAGGTGTAGCGGTGAAATGCGTAGAGATCTGGAGGAACATCAGTGGCGAA
GGCGACTCCCTGGCCAAAGACTGACGCTCATGTGCGAAAGTGTGGGTAGCGAACAGGATTAGATACCCTGGTAGTCCACACCGTAAACGCTGTCTACT
AGCTGTTTGTGATTTTAAATCGTGAGTAGCGAAGCTAACGCGCTAAGTAGACCGCCTGGGGAGTACGGCCGCAAGGTTA

[9]

TACGGAGGGTGCGAGCGTTAATCGGAATTACTGGGCGTAAAGCGCACGCAGGCGGATTGTTAAGCTAGAGGTGAAAGCCCCGCGCTCAACGTGGGAAT
TGCCTTTAGAACTGGCAGTCTAGAGTCTTGGAGAGGGGAGTGGAATTCCAGGTGTAGCGGTGAAATGCGTAGAGATCTGGAGGAACATCAGTGGCGAA
GGCGACTCCCTGGCCAAAGACTGACGCTCATGTGCGAAAGTGTGGGTAGCGAACAGGATTAGATACCCTGGTAGTCCACACCGTAAACGCTGTCTACT
AGCTGTTTGTGTATTTAATACGTGAGTAGCGAAGCTAACGCGCTAAGTAGACCGCCTGGGGAGTACGGCCGCAAGGTTA

References

1. Parada AE, Needham DM, Fuhrman JA. 2016 Every base matters: Assessing small subunit rRNA primers for marine microbiomes with mock communities, time series and global field samples. *Environ. Microbiol.* **18**, 1403–1414. (doi:10.1111/1462-2920.13023)
2. Quince C, Lanzen A, Davenport RJ, Turnbaugh PJ. 2011 Removing Noise From Pyrosequenced Amplicons. *BMC Bioinformatics* **12**, 38. (doi:10.1186/1471-2105-12-38)
3. Oberbeckmann S, Kreikemeyer B, Labrenz M. 2018 Environmental factors support the formation of specific bacterial assemblages on microplastics. *Front. Microbiol.* **8**, 2709. (doi:10.3389/FMICB.2017.02709)
4. De Tender C, Devriese LI, Haegeman A, Maes S, Vangeyte J, Cattrijsse A, Dawyndt P, Ruttink T. 2017 Temporal Dynamics of Bacterial and Fungal Colonization on Plastic Debris in the North Sea. *Environ. Sci. Technol.* **51**, 7350−7360. (doi:10.1021/acs.est.7b00697)
5. Zettler ER, Mincer TJ, Amaral-zettler LA. 2013 Life in the "Plastisphere": Microbial Communities on Plastic Marine Debris. *Environ. Sci. Technol.* **47**, 7137−7146.
6. Kirstein I V., Wichels A, Krohne G, Gerdts G. 2018 Mature biofilm communities on synthetic polymers in seawater - Specific or general? *Mar. Environ. Res.* **142**, 147–154. (doi:10.1016/j.marenvres.2018.09.028)
7. Berry D, Gutierrez T. 2017 Evaluating the detection of hydrocarbon-degrading bacteria in 16S rRNA gene sequencing surveys. *Front. Microbiol.* **8**, 1–6. (doi:10.3389/fmicb.2017.00896)
